# Automated Text Messaging as an Adjunct to Cognitive Behavioral Therapy for Depression: A Clinical Trial

**DOI:** 10.2196/jmir.6914

**Published:** 2017-05-08

**Authors:** Adrian Aguilera, Emma Bruehlman-Senecal, Orianna Demasi, Patricia Avila

**Affiliations:** ^1^ School of Social Welfare University of California, Berkeley Berkeley, CA United States; ^2^ Zuckerberg San Francisco General Hospital Department of Psychiatry University of California, San Francisco San Francisco, CA United States; ^3^ Department of Electrical Engineering and Computer Sciences University of California, Berkeley Berkeley, CA United States

**Keywords:** depression, text messaging, cognitive behavioral therapy, mhealth, mental health, Latinos

## Abstract

**Background:**

Cognitive Behavioral Therapy (CBT) for depression is efficacious, but effectiveness is limited when implemented in low-income settings due to engagement difficulties including nonadherence with skill-building homework and early discontinuation of treatment. Automated messaging can be used in clinical settings to increase dosage of depression treatment and encourage sustained engagement with psychotherapy.

**Objectives:**

The aim of this study was to test whether a text messaging adjunct (mood monitoring text messages, treatment-related text messages, and a clinician dashboard to display patient data) increases engagement and improves clinical outcomes in a group CBT treatment for depression. Specifically, we aim to assess whether the text messaging adjunct led to an increase in group therapy sessions attended, an increase in duration of therapy attended, and reductions in Patient Health Questionnaire-9 item (PHQ-9) symptoms compared with the control condition of standard group CBT in a sample of low-income Spanish speaking Latino patients.

**Methods:**

Patients in an outpatient behavioral health clinic were assigned to standard group CBT for depression (control condition; n=40) or the same treatment with the addition of a text messaging adjunct (n=45). The adjunct consisted of a daily mood monitoring message, a daily message reiterating the theme of that week’s content, and medication and appointment reminders. Mood data and qualitative responses were sent to a Web-based platform (HealthySMS) for review by the therapist and displayed in session as a tool for teaching CBT skills.

**Results:**

Intent-to-treat analyses on therapy attendance during 16 sessions of weekly therapy found that patients assigned to the text messaging adjunct stayed in therapy significantly longer (median of 13.5 weeks before dropping out) than patients assigned to the control condition (median of 3 weeks before dropping out; Wilcoxon-Mann-Whitney *z*=−2.21, *P*=.03). Patients assigned to the text messaging adjunct also generally attended more sessions (median=6 sessions) during this period than patients assigned to the control condition (median =2.5 sessions), but the effect was not significant (Wilcoxon-Mann-Whitney *z*=−1.65, *P*=.10). Both patients assigned to the text messaging adjunct (*B*=−.29, 95% CI −0.38 to −0.19, *z*=−5.80, *P*<.001) and patients assigned to the control conditions (*B*=−.20, 95% CI −0.32 to −0.07, *z*=−3.12, *P*=.002) experienced significant decreases in depressive symptom severity over the course of treatment; however, the conditions did not significantly differ in their degree of symptom reduction.

**Conclusions:**

This study provides support for automated text messaging as a tool to sustain engagement in CBT for depression over time. There were no differences in depression outcomes between conditions, but this may be influenced by low follow-up rates of patients who dropped out of treatment.

## Introduction

### Background

Cognitive behavioral therapy (CBT) is an efficacious treatment for depression [1] delivered via various mediums (eg, individual, group, telephone, Internet) and with diverse populations [2,3]. Low-income and Latino populations can benefit from CBT for depression, but they utilize services at lower rates [4] and have shown lower levels of engagement (homework completion and attendance) once in treatment [5]. Research has shown that increased dosage of psychotherapeutic treatment via increased engagement leads to improved outcomes [6]. Given this literature, research exploring more effective methods of improving attendance of psychotherapy and sustaining engagement with treatment is needed, especially within low income, ethnic minority populations where the problem of nonattendance and dropout is particularly pronounced [7].

### Mobile Messaging to Improve Engagement

Given the association between engagement and improved depression outcomes [6,8,9], interventions that increase engagement in psychotherapy could improve the effectiveness of CBT for depression, particularly in public sector settings. Mobile health (mHealth) tools such as text messaging can increase engagement in psychotherapeutic interventions in a number of ways. Text messaging is low cost and pervasive across socioeconomic and demographic groups in the United States. The delivery of text messages to patients in treatment can prompt engagement in CBT homework, increasing the application of skills learned in therapy sessions, and leading to improved outcomes and treatment adherence [10-12]. The most comprehensive review of text messaging and mental health studies recently concluded that texting is viewed positively and improves adherence and symptom measurement in treatments [12]. Automated text messages during treatment can also serve to “stay on patients’ radars” and make patients feel supported and close to the group, strengthening therapeutic alliance, and increasing the likelihood that they will attend session and reengage with psychotherapy after a period of absence [13,14]. Text messages can further be used to send direct reminders to attend sessions and to take medications. Additionally, data obtained from text message inquiries can be visualized to help behavioral health clinicians provide higher quality, more personalized care. By periodically reviewing graphical representations of feedback on patient progress before session, clinicians can identify key events and address any clinically relevant events during or between sessions [15-17]. As a result of reminding patients about sessions, making patients feel more supported, and making sessions more immediately relevant, mHealth adjuncts to treatment may increase the number of psychotherapy sessions that patients attend and the likelihood that patients will reengage with psychotherapy after a period of absence.

Although studies suggest that patients find mobile technology adjuncts to treatment acceptable and useful [13,15], the impact of text messaging as an adjunct to depression treatment has shown mixed results. One study found that nonautomated text messaging support, in addition to telephone-based psychotherapy, was not related to outcomes (eg, reductions in depressive symptoms or psychotherapy attendance) [18]. In another study, patients with mood and anxiety disorders were randomized to receive psychotherapy appointment reminder texts or no appointment reminders. Although receipt of reminder texts failed to decrease overall rates of psychotherapy nonattendance, patients receiving these texts were less likely to be categorized by their therapists as having prematurely dropped out of psychotherapy [19]. A third study sent messages to women with postpartum depression, but did not report the impact of messaging on outcomes [20]. Agyapong [21] sent supportive text messages to individuals in an inpatient alcohol use disorder and comorbid depression program, and found that patients receiving supportive text messages experienced lower posttreatment Beck Depression Inventory (BDI) scores than those receiving treatment-as-usual, but the effects did not hold 3 months after text messages were terminated. Although studies have sought to assess differences in key symptom outcomes, they have less often assessed impacts on engagement in mental health interventions. Even if outcomes are not significantly improved with the addition of an mHealth adjunct, engagement may improve, thereby providing more benefit to more people by decreasing attrition.

### Aim of This Study

The aim of this study was to test whether a text messaging adjunct (mood monitoring text messages, treatment-related text messages, and a clinician dashboard to display patient data) increases engagement and improves clinical outcomes in a group CBT treatment for depression. Specifically, we aim to assess whether the text messaging adjunct led to an increase in group therapy sessions attended, an increase in duration of therapy attended, and reductions in Patient Health Questionnaire-9 item (PHQ-9) symptoms compared with the control condition of standard group CBT in a sample of low-income Spanish speaking Latino patients. We expect that receiving text messages would result in higher attendance and improved depression outcomes.

## Methods

### Recruitment

Patients utilizing outpatient services at an urban public hospital were referred to a behavioral health clinician by their primary care provider when there were concerns about depression due to qualitative symptom expression or a positive screen based on the PHQ-9 [22], a commonly used depression measure in primary care. Patients were seen by a behavioral health clinician following their primary care visit or contacted by phone if seen after hours. Patients were considered eligible for group therapy for depression if they had a PHQ-9 score of 10 or above at the time of initial assessment by the behavioral health clinician. Exclusion criteria for group treatment were active suicidal ideation with a plan and active, severe psychosis. The behavioral health clinician provided a brief behavioral intervention and a referral to group CBT if the patient met the above criteria.

This study used a nonrandomized design to allocate patients into a texting intervention or nontexting control group. Patients were administered consent, baseline questionnaires, and enrolled in the study during each patient’s first group therapy session attended, therefore data for patients eligible but not interested in or declining treatment is unavailable. We intended to conduct a randomized controlled trial (RCT), but we encountered organizational and patient level barriers similar to barriers in other projects in low-income public sector settings [23]. At the organizational level, study materials, such as a paper or an online randomization table, were not always accessible to clinicians. At the patient level, scheduling challenges often precluded patients from attending at the day and time they were assigned. Since the intervention was an adjunct to standard care, it was not possible to deny treatment if a patient was not able to attend the group to which they were initially randomized and were instead allowed to attend the group that fit best with their schedule. Additionally, referring clinicians assigned patients to groups based on the need to balance group size, which may impact group dynamics [24].

Patients were neither incentivized to start treatment, attend sessions, nor complete surveys. Neither the therapists and patients nor research assistants were blinded since they participated in the delivery of treatment and data collection. All procedures and materials were approved by the University of California, San Francisco Institutional Review Board Committee.

### Intervention

#### Group Cognitive Behavioral Therapy

Patients referred to group CBT were allocated to one of the two study conditions: group CBT without the text messaging adjunct or group CBT with the text messaging adjunct. Patients in both study conditions participated in a weekly manualized cognitive behavioral group treatment based on an adapted version of the Building Recovery by Improving Goals, Habits, and Thoughts (BRIGHT) manual for depression [25]. The treatment manual was developed in English and Spanish for use in public sectors settings and has been found to be an efficacious treatment for depression in this population [26]. The manual is divided into four, four-week modules largely focusing on cognitive restructuring (thoughts), behavioral activation (activities), interpersonal relationships (people), and healthy behaviors (health). Treatment was delivered by two therapists at a time (total of two clinical psychologists, and two licensed clinical social workers). Therapists in both conditions delivered culturally sensitive care, leading the groups in Spanish, and all had years of experience providing therapy to low-income Latinos.

Psychotherapy was structured as two continuously running groups (a texting and a control group), and was designed to last 16 continuous weeks for each patient, with group sessions offered weekly. Patients were admitted to the groups on a rolling basis to minimize wait times. Group size in any given week varied from as few as 1 patient to as many as 8, with a median group size of 3 patients. Although the manual was structured for 16 weeks of therapy, some patients attended for more than 16 sessions, or longer than a 16-week period, if they began mid-module or had an extended absence in the middle of treatment. However, our analyses focus only on the 16-week period after initial participation in treatment, as this time frame represented potential completion of all content within the BRIGHT manual.

Both conditions used the same BRIGHT manual and differed only in the exclusion or inclusion of paper-based, weekly mood monitoring worksheets and skill-building homework projects. While the nontexting manual included the paper-based homework sheets, patients in the texting condition were instead sent a series of text messages for mood tracking and skill-building in order to complete “real-world” practice.

#### Text Messaging Adjunct

**Figure 1:**
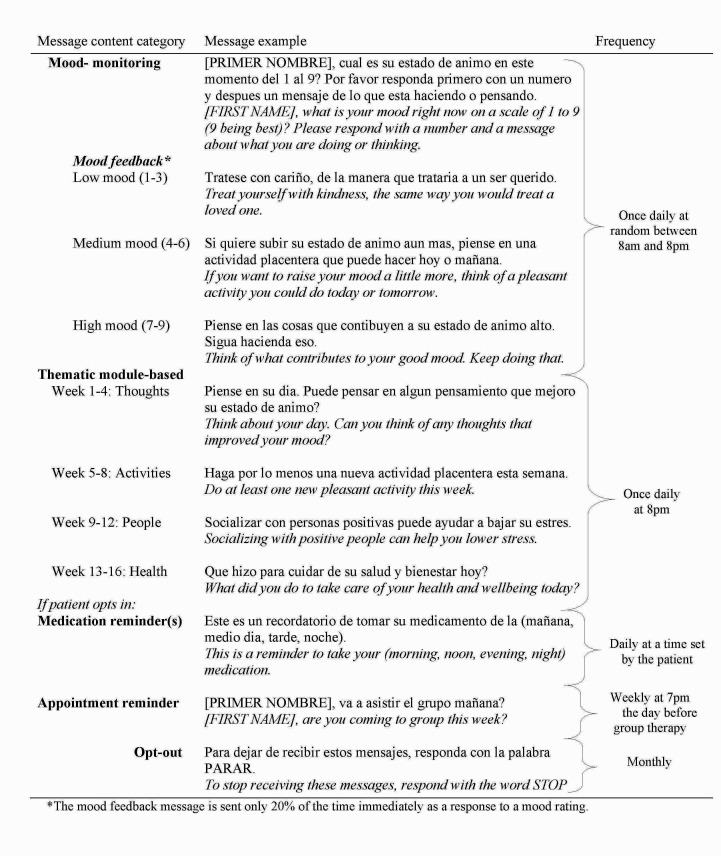
Sample text messages received by patients in the texting condition during depression treatment translated to English.

Patients were instructed on how to reply to the messages upon enrollment. Patients in the texting condition received up to five types of automated text messages (detailed in Figure 1): (1) a daily mood rating prompt (and feedback 20% of the time based on their mood ratings), (2) a daily message supplementing live therapy content (ie, a thematic module-based message), (3) optional daily medication reminders, (4) a weekly reminder to attend psychotherapy, and (5) a monthly opt-out message to terminate message delivery, if desired. Messages were automatically delivered to patients on a predetermined schedule, with the exception of patients deciding whether and when to receive medication reminders. All text messages were delivered in Spanish (see Figure 1).

The daily mood rating prompt was an integral part of the intervention designed to promote mood-monitoring and increase mood state awareness, a critical element of depression treatment [27]. Automated mood rating messages also allowed for the collection of real-time data in each patient’s daily environment for a clinician to review. Patients were given feedback on their mood responses a random 20% of the time they sent a mood response (see Figure 1).

An additional daily text message was delivered reinforcing the theme of the therapy session that week (ie, a thematic module-based message). The content for this message was developed from the BRIGHT manual [25] and focused generally on cognition, self-monitoring, behavioral activation, interpersonal interactions, and healthy behaviors affecting mood.

Patients in the texting condition received up to two types of reminder messages—optional daily medication reminders and weekly reminders to attend group psychotherapy appointments, the content of which is described in Figure 1. Additionally, patients in both conditions were reminded to attend therapy by phone when they began and thereafter only when group sessions were canceled, such as during holidays.

**Figure 2:**
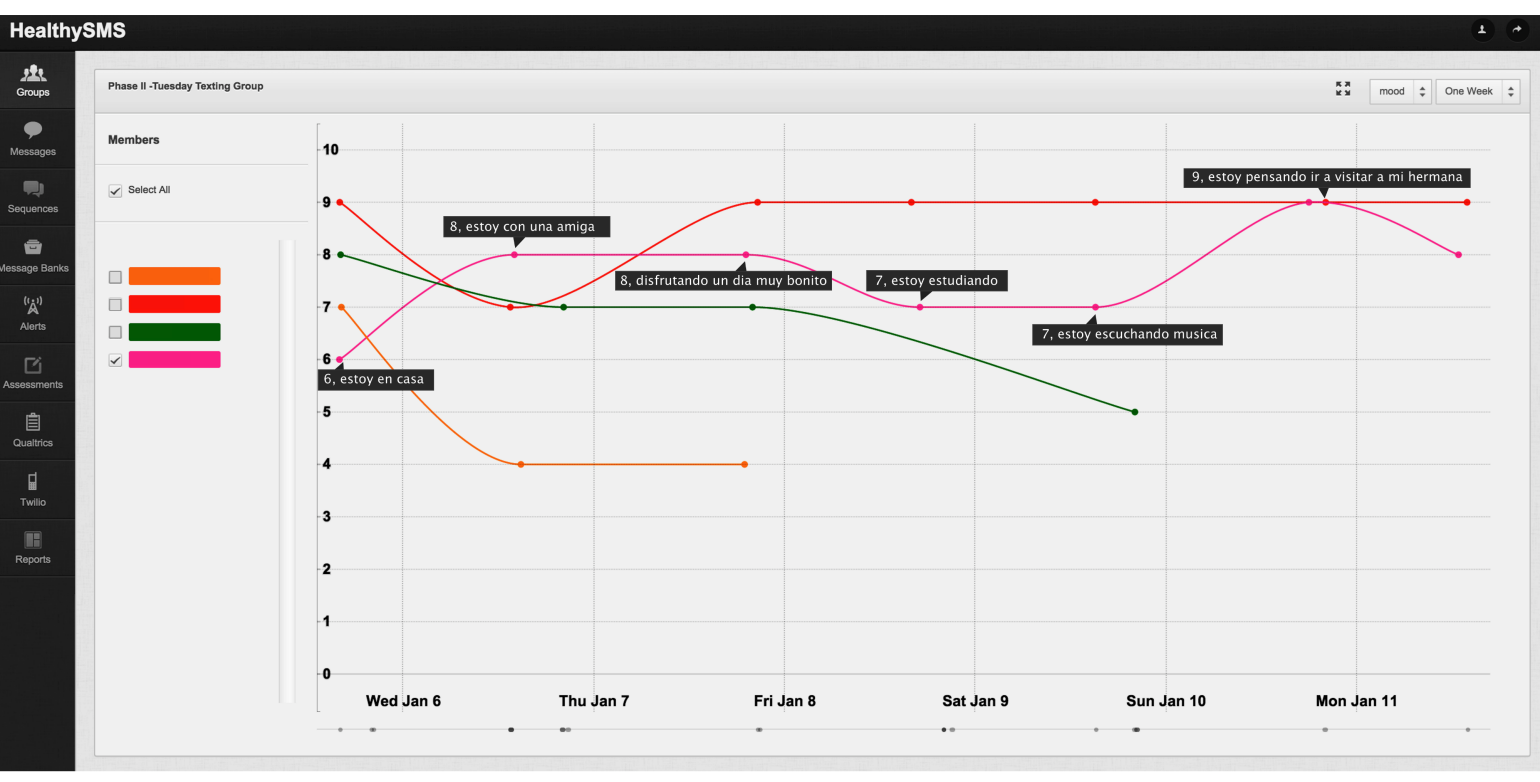
Sample mood graph from HealthySMS used to review in between session mood.

Messages were delivered through an automated text messaging platform, HealthySMS developed for the study by the first author (Multimedia Appendix 1). HealthySMS was designed as a tool for clinicians delivering therapeutic interventions to schedule automated and real-time message delivery. The platform allows clinicians to track patient progress during treatment and monitor responses to the text message prompts in between sessions (Figure 2). In particular, patient mood data from HealthySMS was projected onto a whiteboard at the beginning of each therapy session to assess patients’ mood states in the past week and to apply the tools of therapy to specific events with an emphasis on low and high points.

Messages were sent to patients after terminating therapy for 6 additional months or until they opted out. The content delivered after treatment was equivalent to the content during treatment differing only in the randomization of message order. Patients were instructed that they could opt-out of text messaging at any time by texting the word “STOP” or “PARAR,” in Spanish, or by notifying a staff member.

#### Phases and Iterations of the Intervention

**Figure 3:**
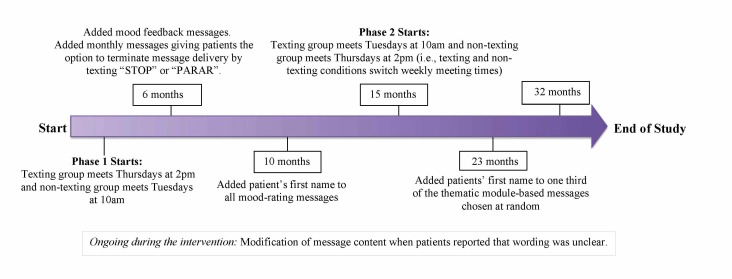
Iterations made to the text message content during the intervention.

Group therapy sessions held earlier in the day may be more difficult for patients to attend due to depression symptoms, patient characteristics, or employment, therefore two phases (Phase 1 and Phase 2) of the study were conducted in order to switch the day of week and time of day at which the texting and control condition groups took place. In Phase 1 of the study, the texting group (n=21) was held on Thursday afternoons at 2pm and the control group (n=25) was held on Tuesday mornings at 10am (see Figure 3). In Phase 2, the conditions switched meeting times, with the texting group meeting on Tuesday mornings (n=24), and the control group meeting on Thursday afternoons (n=15). Patients attended psychotherapy in only one of the two phases of the study, so each patient attended psychotherapy on only one day of the week, and was exposed to only one treatment condition. Although the same primary therapist (a clinical psychologist) conducted all groups, the cotherapist in both phases of the study differed.

Iterations were also made to improve the text messaging program (Figure 3) based on ongoing feedback from patients and knowledge acquired from emerging mHealth research aimed at improving usability [28]. None of the changes impacted the core elements of CBT or purpose of the study. This approach allowed for the continuous incorporation of knowledge acquired during the intervention, increasingly considered important in research involving behavioral intervention technologies [29,30].

There were no adverse events reported during the study. The HealthySMS platform scanned messages for indications of suicidal ideation by identifying keywords (eg, suicide, kill, die, jump, bridge, and so on) and would notify the principal investigator if any keywords appeared. In total, 24 instances of alerts were triggered, but none of the messages indicated actual suicidal ideation.

### Measures

Participants completed a baseline questionnaire at their first therapy session that collected demographic data, familiarity with mobile phone use, and measures assessing depression and anxiety symptoms, medication adherence, and depression self-efficacy. For the purposes of this study, only demographic and mobile phone use variables, as well as depression symptoms, are reported.

#### Attendance

Attendance was recorded at each scheduled group session. Attendance was coded as 1 if the patient was present at the session and 0 if they did not attend. Two key measures of attendance were derived: (1) the total number of psychotherapy sessions patients attended in the first 16 weeks of therapy and (2) the number of weeks patients stayed in treatment before dropping out, up the 16th session of treatment. We included attendance data up until the 16th session of treatment, as this time frame represented potential completion of all content within the treatment manual. The total number of psychotherapy sessions attended was calculated by taking the simple sum of the sessions patients attended over the 16-session period. The number of weeks patients stayed in treatment was calculated by counting the number of sessions that elapsed between the first and the last session the patient attended in this same time frame. A patient was considered to have dropped out of therapy when he or she failed to return to any future therapy session offered.

#### Depressive Symptoms

Depressive symptoms were measured using the 9-item Patient Health Questionnaire [22,31]. The reliability and validity of the PHQ-9 has been demonstrated in Latino samples [32]. The PHQ-9 was administered at baseline and at every session that a patient attended. Therapists and a research assistant aided patients in completing the questionnaires, first by using paper and pencil measures and later by using a digital equivalent on an iPad. The PHQ-9 was not administered when patients missed a therapy session.

### Analytic Plan

Given the inability to randomize to condition, we first tested whether the texting and control conditions differed on any key characteristics at baseline, including depressive symptom severity, familiarity with mobile technologies, and basic demographic characteristics. We then tested the central research questions—whether the conditions differed in their total number of sessions attended, time until dropout, and degree of depressive symptom recovery over the first 16 weeks of treatment.

Condition differences in total sessions attended and weeks in treatment before dropout from psychotherapy were tested using Wilcoxon-Mann-Whitney *U* test for independent samples with patients stratified by study phase. This nonparametric approach was chosen to avoid normality assumptions on the underlying distributions, given that the distributions of the attendance variables were nonnormal. Because there was an unequal distribution of patients to condition across the two study phases, we stratified by phase to conservatively control for any differences that may have been confounding between the study phases. Statistical analyses were performed using the asymptotic assumption on the test statistics [33].

Condition differences in depressive symptom recovery were tested using linear mixed models. The data consisted of repeated measures of the PHQ-9 (up to 16, corresponding to the 16 weeks of therapy), nested within patients. To model dependencies in the same patient’s PHQ-9 scores across time, we used a two-level hierarchical model, modeling patient-specific (ie, random) intercepts and patient-specific slopes for week of therapy. The model included condition, week of therapy, and the interaction of condition and week of therapy as predictors, and weekly PHQ-9 scores as the outcome. The condition×week of therapy term allowed for an assessment of whether the texting condition experienced greater depressive symptom improvement over the course of therapy than the control condition. As with the attendance outcomes, we also controlled for study phase to conservatively control for confounds linked to this variable. Analyses utilized all data points for participants (*j*) and occasions (*i*), where neither the response *y*_ij_ (ie, weekly PHQ-9 scores) nor the covariates *x*_ij_ were missing. For patients who dropped out of therapy before their 16th session, all and only those data collected before the date of attrition were included in these models.

Initial analyses utilized an intent-to-treat approach, including all patients, with the exception of patients who had participated in prior group CBT studies in the clinic. These “returning” patients were excluded from the analyses due to concern that their prior familiarity with the treatment and texting protocol might bias their data. We compared these intent-to-treat findings with results of “active texters.” Active texters refers only to patients who utilized the texting adjunct at least once (we excluded the data of patients assigned to the texting condition who either did not receive any texts, or did not respond to any texts received during the first 16 weeks of therapy). To differentiate these analyses from the intent-to-treat analyses, we refer to them as “active-texting” analyses. Active texting analyses were conducted to better isolate the potential effect of the texting adjunct with actual users of the intervention. Patients included in the control condition remained constant over both the intent-to-treat and the active-texting analyses. All statistical analyses were performed using Stata—the attendance analyses were implemented with the vanelteren package for conducting stratified Wilcoxon-Mann-Whitney *U* test, and the depression analyses were implemented via the xtmixed command.

## Results

### Participants

A total of 91 patients were enrolled in the study between January 2014 and August 2016, of which 48 were allocated to the texting condition and 43 were allocated to the control condition. We excluded 6 patients (3 from each condition) from all analyses because they had participated in prior group CBT studies within this same behavioral health clinic. This exclusion resulted in 85 patients, 45 in the texting condition, and 40 in the control. A total of 6 patients in the texting condition were further excluded from the active-texting analyses, 2 because they sent no text messages during the first 16 weeks of therapy, and another 4 because they did not respond to a single message received during this period. This additional exclusion resulted in 39 active texting patients in the texting condition, and 40 in the control.

### Baseline Data

The baseline characteristics of patients (Table 1) did not differ significantly between the two conditions. Patients were predominantly female and middle aged, with a relatively low level of education (81% of patients [68/84] lacked a high school diploma or equivalent). Slightly less than half of all patients reported being in therapy for depression in the past (44%, 37/85) and similar numbers reported taking medication for depression at baseline (45%, 38/85). The majority of patients in both conditions owned a mobile phone (94%, 80/85), and were familiar with how to use text messaging (73%, 57/78).

The following variables have missing data for 1-2 patients—marital status, education, use of SMS in prior month, preferred method of contact.

### Outcomes

#### Condition Differences in Attendance Patterns

For the attendance analyses, an additional 3 patients (1 in the intervention and 2 in the control) were excluded, because the study ended before these patients had been offered 16 sessions of therapy. Thus, the total number of sessions attended by these three patients and their time in therapy before dropout was not comparable to that of other patients. This exclusion resulted in 82 patients in the intent-to-treat analyses (44 in the texting condition and 38 in the control) and 76 patients in the active-texting analyses (with 38 patients in each condition).

##### Total Sessions Attended

**Figure 4:**
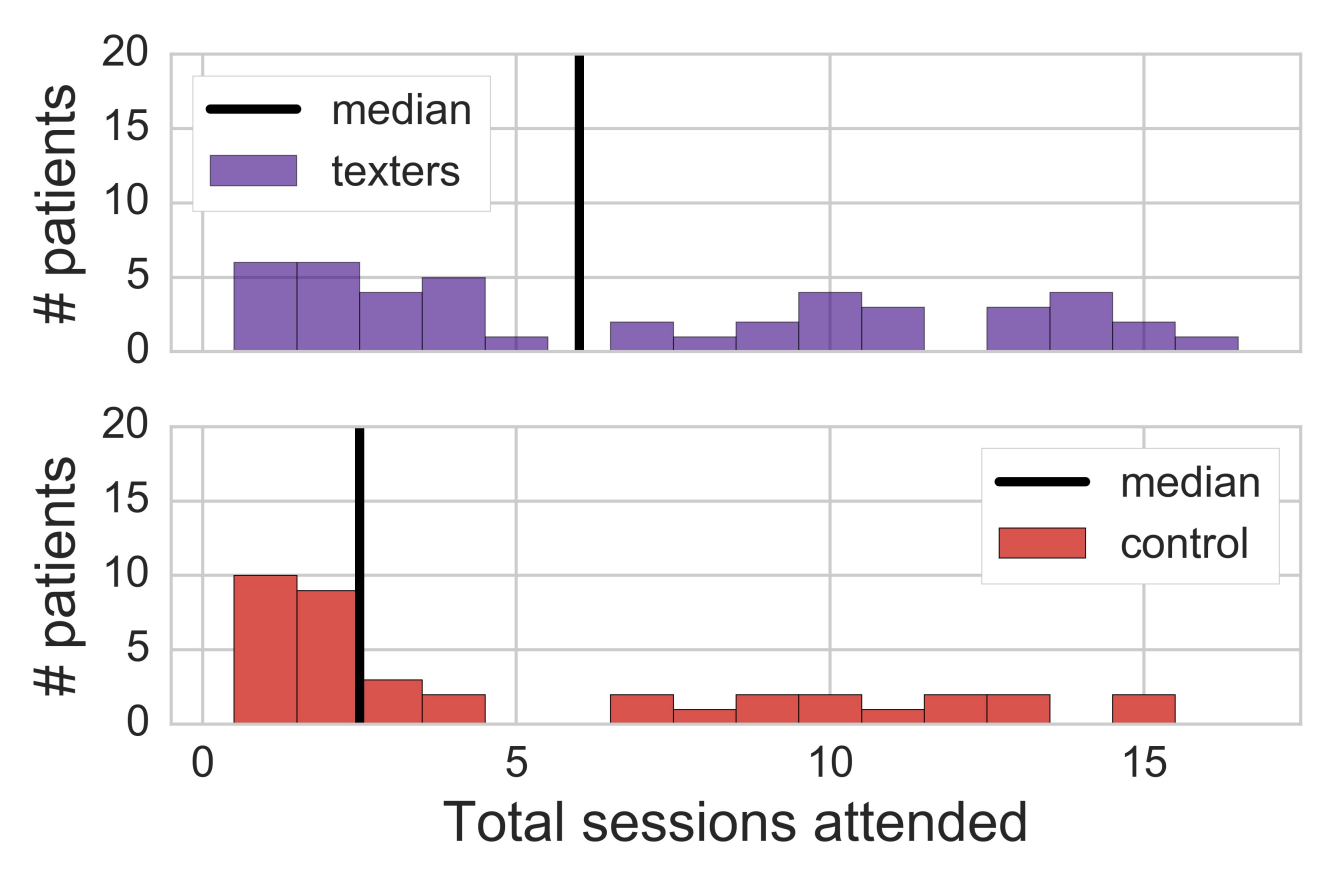
Condition differences in total sessions attended. Figures display intent-to-treat analyses.

Intent-to-treat analyses indicated that patients assigned to the texting condition generally attended more sessions across the first 16 weeks of therapy than patients assigned to the control condition, but the effect was not statistically significant when patients were stratified by phase (Wilcoxon-Mann-Whitney, *z*=−1.65, *P*=.10). The median number of sessions attended by patients assigned to the texting condition was 6, whereas the median number of sessions attended by control patients was 2.5 (Figure 4). The effect was marginally significant when only the data of active texters was compared with that of the control condition (Wilcoxon-Mann-Whitney, *z*=−1.91, *P*=.06), with active texters attending a median of 7.5 sessions in the first 16 weeks of therapy.

**Table 1:** Baseline characteristics in the texting and control conditions. Statistics reflect intent-to-treat analyses.

Characteristics		Texting condition (n=45)	Control condition (n=40)	*P* value
**Age (years), mean (SD^a^)**		51.71 (11.55)	51.83 (11.73)	.96^b^
**Gender: female, n (%)**		38 (84.44)	29 (72.50)	.18^c^
**Highest education, n (%)**				.11^c^
	No formal education	3 (6.67)	4 (10.26)	
	1st to 5th grade	12 (26.67)	7 (17.95)	
	6th to 8th grade	12 (26.67)	12 (30.77)	
	Some high school	14 (31.11)	4 (10.26)	
	High school grad or GED^d^	2 (4.44)	3 (7.69)	
	Some college	1 (2.22)	6 (15.38)	
	College graduate	1 (2.22)	2 (5.13)	
	Graduate or professional school	0 (0.00)	1 (2.56)	
**Employment status, n (%)**				.73^c^
	Full-time	7 (15.56)	4 (10.00)	
	Part-time	7 (15.56)	4 (10.00)	
	Homemaker	3 (6.67)	3 (7.50)	
	Unemployed	12 (26.67)	13 (32.50)	
	On disability	10 (22.22)	12 (30.00)	
	Retired	4 (8.89)	4 (10.00)	
	Other	2 (4.44)	0 (0.00)	
**Marital status, n (%)**				.76^c^
	Single	17 (38.64)	14 (35.00)	
	Married or partnered	13 (29.55)	13 (32.50)	
	Divorced or separated	7 (15.91)	9 (22.50)	
	Widowed	7 (15.91)	4 (10.00)	
**Depression measures**				
	PHQ-9^e^, mean (SD)	13.36 (5.96)	13.13 (4.99)	.85^b^
	Prior therapy for depression, n yes (%)	21 (46.67)	16 (40.00)	.54^c^
	Medication for depression, n yes (%)	22 (48.89)	16 (40.00)	.41^c^
	Prior hospitalization for depression, n yes (%)	5 (11.36)	7 (17.50)	.42^c^
**Mobile phone ownership, n yes (%)**		43 (95.56)	37 (92.50)	.55^c^
**Use of text-messaging in prior month, n yes (%)^g^**		34 (79.07)	23 (65.71)	.19^c^
**Preferred method of contact^f^, n (%)**				.74^c^
	Call	27 (61.36)	26 (66.67)	
	Text	6 (13.64)	6 (15.38)	
	Depends	11 (25.00)	7 (17.95)	

^a^SD: standard deviation.

^b^Indicates that a *t* test was used to test for condition differences.

^c^Indicates that a chi-square test was used to test for condition differences.

^d^GED: general educational development.

^e^PHQ-9: Patient Health Questionnaire-9 item.

^f^Preferred method of contact refers to the question “In general, if someone needs to reach you, do you prefer that they call or that they text you?”

^g^Question asked only to the patients who reported owning a mobile phone.

##### Time Until Dropout

**Figure 5:**
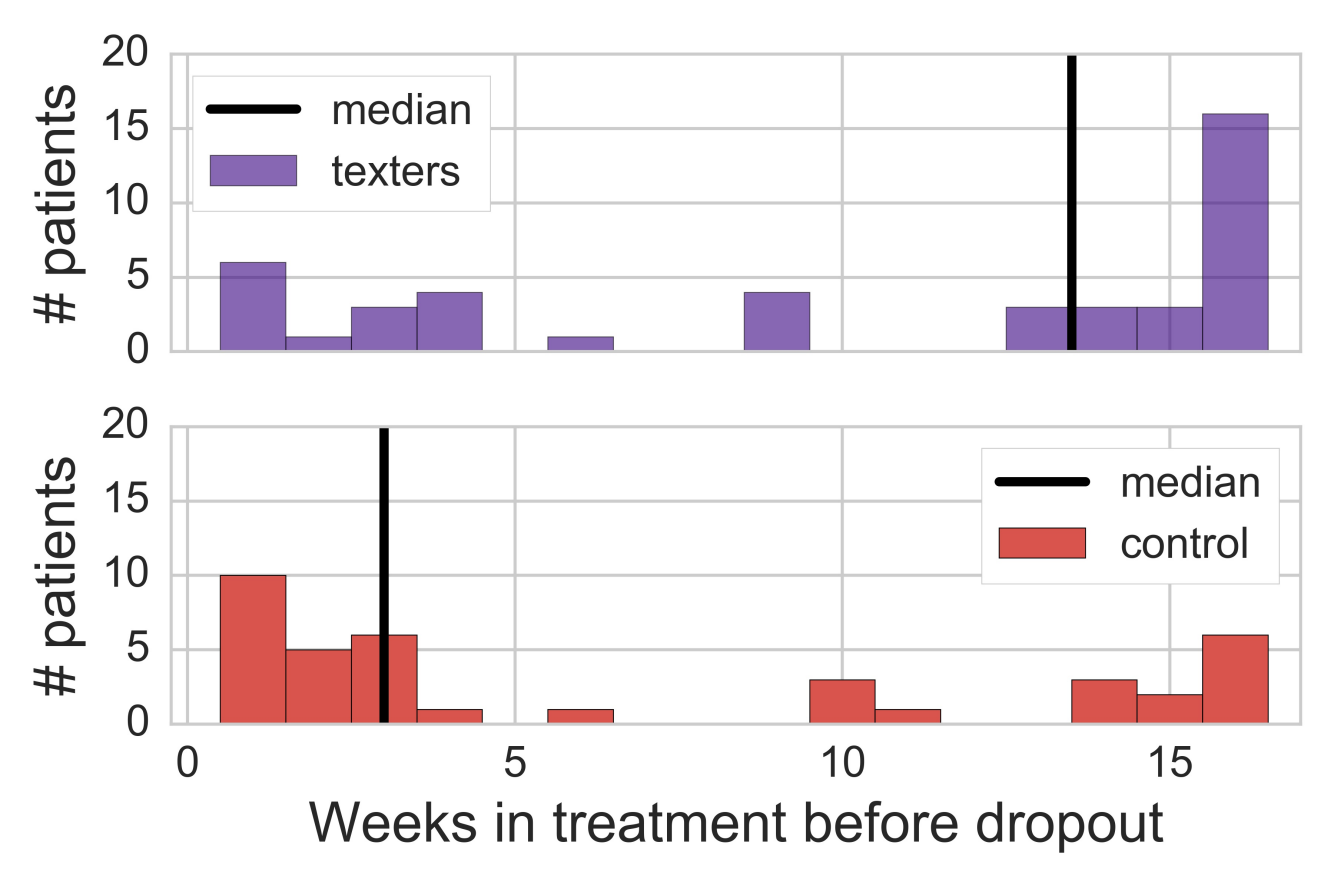
Condition differences in weeks in treatment until patient dropout. Figures display intent-to-treat analyses.

Intent-to-treat analyses indicated that patients assigned to the texting condition stayed in therapy significantly longer before dropping out than patients assigned to the control condition when stratified by phase (Wilcoxon-Mann-Whitney, *z*=−2.21, *P*=.03). Patients assigned to the texting condition stayed in therapy for a median of 13.5 weeks before dropping out, whereas patients in the control conditions stayed in therapy for a median of only 3 weeks before dropping out (Figure 5). Results were substantively similar when contrasting the duration data of active texters (median of 14 weeks until dropout) to that of patients in the control condition (Wilcoxon-Mann-Whitney, *z*=−2.28 *, P*= *.* 02).

#### Condition Differences in Depression Symptom Recovery

Intent-to-treat analyses indicated that, as anticipated, depressive symptoms significantly declined in both the texting condition (*B*=−.29, 95% CI −0.38 to −0.19, *z*=−5.80, *P*<.001) and the control condition (*B*=−.20, 95% CI −0.32 to −0.07, *z*=−3.12, *P*=.002) over the first 16 weeks of therapy. These coefficients indicate that, on average, the texting condition’s PHQ-9 scores decreased 0.29 points for every week that patients were enrolled in therapy, whereas the control condition’s PHQ-9 scores decreased an average of 0.20 points per week. However, the magnitude of depressive symptom improvement in the texting condition was not significantly greater than that of the control (*B* of the condition×therapy week interaction=−.09, 95% CI −0.25 to 0.07, *z*=−1.09, *P*=.27). Similarly, active texting analyses failed to demonstrate greater improvements in depressive symptom recovery among active texters as compared with the control condition (*B* of interaction=−.10, 95% CI −0.27 to 0.06, *z*=−1.25, *P*=.21). Descriptive statistics on PHQ-9 scores by condition corresponding to each the 16 psychotherapy sessions are provided in Table 2.

**Table 2:** Means, standard deviations, and sample size for the Patient Health Questionnaire-9 item (PHQ-9) by condition across psychotherapy. Statistics reflect intent-to-treat analyses.

	Texting condition Mean		Control condition Mean	
Psychotherapy session	PhQ-9 (SD^a^)	n	PhQ-9 (SD)	n
1 (baseline)	13.36 (5.96)	45	13.13 (4.99)	39
2	9.90 (6.15)	21	10.60 (4.71)	20
3	8.83 (5.31)	23	9.83 (5.60)	18
4	6.94 (5.15)	17	9.07 (4.35)	15
5	9.44 (5.85)	18	9.90 (4.33)	10
6	7.32 (5.61)	19	7.71 (5.31)	14
7	7.56 (5.18)	18	7.85 (5.80)	13
8	6.35 (3.44)	17	10.53 (5.55)	15
9	7.09 (4.12)	23	8.73 (3.29)	11
10	6.83 (6.08)	18	7.93 (6.13)	15
11	7.05 (4.50)	22	7.44 (4.88)	9
12	6.38 (3.84)	13	6.33 (4.85)	9
13	7.84 (6.24)	19	6.80 (4.92)	10
14	5.24 (3.40)	17	9.10 (5.67)	10
15	8.07 (5.74)	15	9.83 (6.55)	6
16	6.00 (4.20)	18	12.67 (6.40)	9

^a^SD: standard deviation.

## Discussion

### Principal Findings

This study investigated whether a mobile phone-based text messaging adjunct to CBT for depression increased engagement and improved clinical outcomes compared with standard CBT without a text messaging adjunct. We found that receiving text messages during a group CBT intervention led to decreased attrition in a sample of Spanish-speaking Latinos with depression. These findings support the use of text messaging and mHealth interventions as adjuncts to psychotherapeutic treatments in order to reduce attrition. Patients in the texting condition may have felt more engaged in the intervention and the messages may have helped them practice skills throughout the week. It is also possible that receiving text messages helped patients feel more supported and more motivated [13], and thus diminished the symptomology inherent of depression by targeting maladaptive thoughts and behaviors [34] that could prevent patients from attending therapy. At the most basic level, it is possible that the simple act of receiving a weekly reminder encouraged patients to resume therapy attendance even after one or several sessions were missed [14]. It is also possible that the review of patient data in HealthySMS allowed clinicians to provide more personalized interventions based on patient mood and text responses that helped keep patients more engaged.

Despite a relatively small sample size, we explored whether there were significant differences in PHQ-9 ratings between conditions over the course of the intervention. We did not find significant differences in PHQ-9 outcomes between the conditions. Since both conditions received an active treatment and the control condition has been found to be effective in previous studies [26], we would need a larger sample size to detect smaller differences in outcomes. It is also possible that people who did not attend were more depressed, but we were unable to assess PHQ-9 ratings when patients did not attend.

Even though we did not find differences in symptoms between conditions, our findings that patients stayed in treatment longer indicate that the text messaging adjunct can promote sustained engagement with an already efficacious treatment. Results from this study may even be applicable to CBT delivered through other modalities such as the Internet, that suffer from high levels of attrition, by bringing the treatment into users’ daily lives. However, attrition is higher and engagement with technology is lower without human support [35]. More research is needed to determine an ideal balance between digital and human intervention.

Other key steps in advancing this area of research would be to determine whether there are broader cost savings by sending automated messages and keeping patients in treatment longer. A key selling point for mHealth technologies is the ability to reduce health care costs [36-38]. Although patients staying in psychotherapy longer is seemingly more costly in the short-term, it is possible that receiving a stronger dose of treatment and having more people complete treatment can improve recovery from depression and reduce associated costs such as losses in productivity and other depression-related societal burdens long-term. This study was conducted in group therapy setting, which is already less resource-intensive than individual therapy, thus the cost reduction associated with a text message adjunct may be greater.

Despite the recent proliferation of interventions using technology-based symptom monitoring via mobile smartphone apps and passive sensing [39], text messages (and other mobile messaging apps) remain a low cost and simple way of collecting data from patients with low technological ability who may otherwise have a difficult time logging symptoms in apps or websites [15,40]. Despite the automation of messages, they also offer a personal connection to another individual, whether it is perceived or real [13].

### Generalizability

The study included low-income, Spanish-speaking Latinos. It is unclear how these findings translate to other populations. Research has shown that this population generally has lower engagement in mental health interventions [5], therefore it was appropriate to test the texting adjunct with this population. A strength of our study is that it occurred as part of standard clinical practice in a patient pool with multiple chronic illnesses that is typically less responsive to interventions and is thus more likely to successfully generalize to other clinical settings. It is likely that our findings in this population that is less educated, less technologically savvy, and more difficult to engage may generalize to more diverse and higher socioeconomic status (SES) populations. It is also possible that mHealth and text messaging adjuncts could improve other individual and group psychotherapies from other modalities or focusing on something other than depression, especially if they rely on completion of between session homework and skills practice.

### Limitations

This study was not fully randomized, despite our initial intention. Furthermore, the lack of randomization opens up the possibility of third variables being responsible for any group differences. However, given that there were no baseline differences between the groups, the possibility of confounds may be low. Another limitation is that clinicians and data collectors were not blind to conditions, which opens up the possibility of bias. Last and most important, we were unable to assess depressive symptoms on days patients missed treatment. This particularly limits the interpretation of no differences in outcomes between the groups. It is possible that people stopped attending either because they were highly symptomatic or vice versa because they felt recovered. Future studies should ensure that assessments are collected regardless of therapy session attendance.

### Future Directions

Future research should more specifically study mechanisms of action to improve the efficiency and effectiveness of mHealth treatment adjuncts. Future studies should also assess the impact of text messaging and mHealth adjuncts on symptoms in a larger sample size to determine if there is clinically significant improvement compared with standard group CBT. They should also make sure to collect symptom data from nonengaged patients. It is possible that gains from therapy can be maintained longer if patients continue to receive text messages that encourage them to practice skills thought to be active ingredients in improvements. Along with research on overall efficacy and effectiveness of mHealth interventions, studies should assess how to utilize incoming data to predict key clinical events. For example, analyses of daily mood data found that lower mood the day before a therapy session resulted in lower likelihood of attendance [41]. These types of analytics can inform just in time interventions to improve mHealth and in person interventions. Finally, cost-effectiveness analyses can help determine the relative value of increasing attendance to psychotherapy sessions and whether that improves outcomes long-term, resulting in lower health care costs overall.

### Conclusions

Our study shows that a text messaging intervention used as an adjunct to psychotherapy for depression can improve engagement in treatment. We found that patients who received text messages dropped out later in treatment compared with patients receiving standard CBT treatment. By testing this intervention in a low-SES Latino population, it may also generalize to other populations that have been difficult to engage in mental health treatment. As the focus of translational science moves to improve the implementation of efficacious interventions for the broad benefit of the public’s health, mobile interventions as adjuncts to treatment are emerging as valuable tools.

## Appendix

Multimedia Appendix 1 Example of clinician dashboard from HealthySMS.

## Abbreviations

BDI: Beck Depression Inventory
CBT: cognitive behavioral therapy
mHealth: mobile health
PCP: primary care provider
PHQ-9: Patient Health Questionnaire-9 item
RCT: randomized controlled trial
SES: socioeconomic status
