# Supplementary figures and images for: Automated Text Messaging as an Adjunct to Cognitive Behavioral Therapy for Depression: A Clinical Trial
Source: J Med Internet Res. 2017 May 8;19(5):e148. doi: 10.2196/jmir.6914 (PMC5440738; doi:10.2196/jmir.6914)

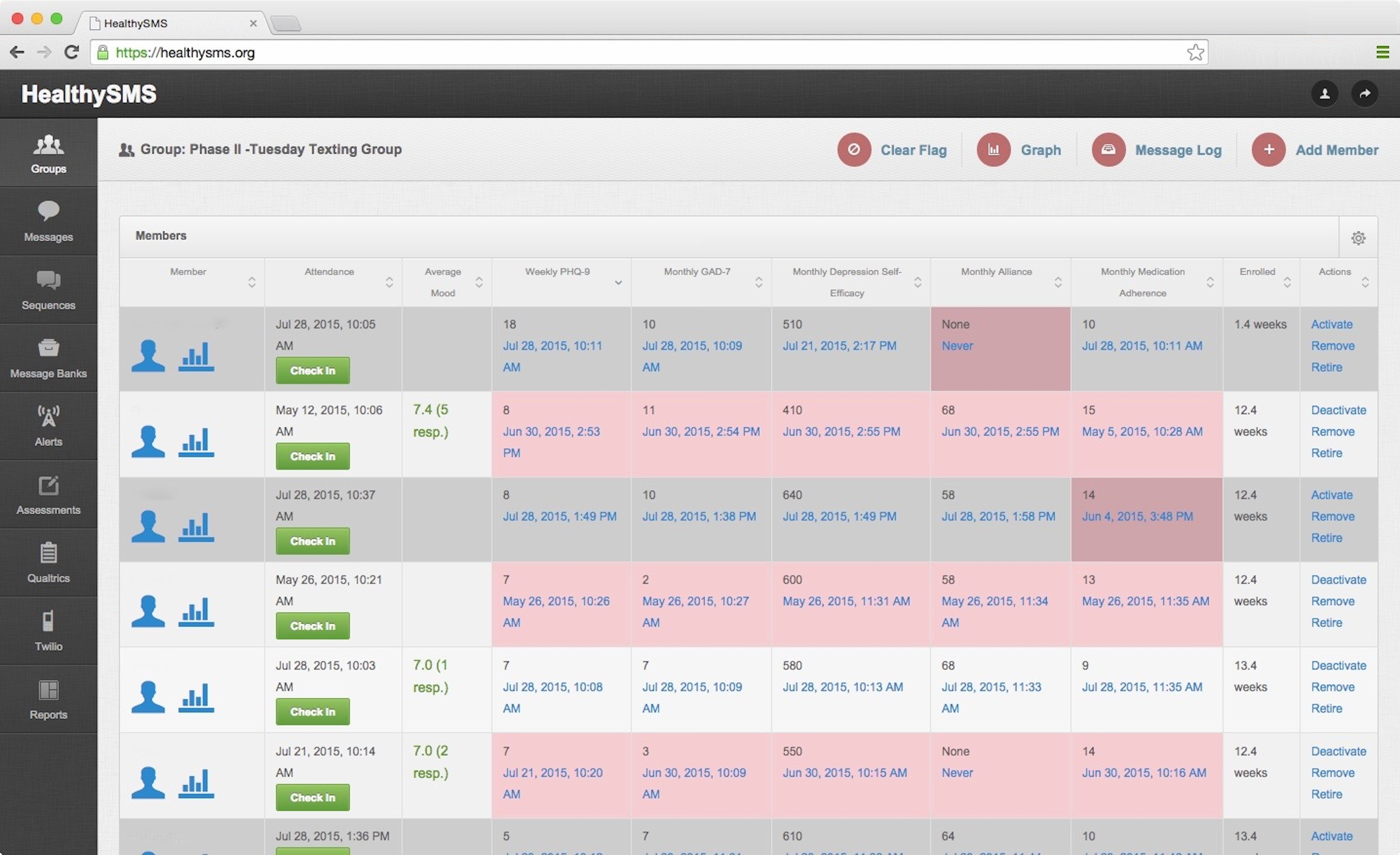

Supplement: Multimedia Appendix 1 [file jmir_v19i5e148_app1.jpeg]
